# Supplementary material for: Expression of RAS and RAB interactor 1 (RIN1) in head and neck tumors at selected hospital in Ghana
Source: PLoS One. 2024 Apr 18;19(4):e0301295. doi: 10.1371/journal.pone.0301295 (PMC11025731; doi:10.1371/journal.pone.0301295)
Supplement: Attachment — Submitted filename: PONE-D-23-28228 (Reviewed).pdf [file pone.0301295.s002.pdf]

PLOS ONE

EXPRESSION OF RAS AND RAB INTERACTOR 1 (RIN1) IN HEAD AND NECK TUMORS AT SELECTED HOSPITAL IN GHANA

--Manuscript Draft--

**Manuscript Number:**	PONE-D-23-28228
**Article Type:**	Research Article
**Full Title:**	EXPRESSION OF RAS AND RAB INTERACTOR 1 (RIN1) IN HEAD AND NECK TUMORS AT SELECTED HOSPITAL IN GHANA
**Short Title:**	HEAD AND NECK TUMORS
**Corresponding Author:**	Precious Barnes, PHD Student<br>University of Cape Coast, Ghana<br>Cape Coast, Cape Coast GHANA
**Keywords:**	Head and neck tumor, oral cavity, RIN1
**Abstract:**	BACKGROUND Head and neck tumor (HNT) are tumor of the paranasal sinuses, the salivary glands and the upper aerodigestive tract. RIN1 is a Ras effector protein regulating epithelial cell properties and has been implicated in a number of cancers. METHOD The aim of this study was to investigate the expression of RIN1 in head and neck tumor. RIN1 expression was analyzed using quantitative real-time PCR (qRT-PCR) and immunohistochemical staining on tissue samples from ~~a~~ consecutive series of 150 head and neck tumor patients who underwent tumor resections between 2014 and 2018. RESULTS The relationship between RIN1 expressions, clinicopathological factors, was investigated. qRT-PCR result[illegible]owed that the RIN1 mRNA expression was low in tumor tissue samples than in t[illegible]1expression were low as compared with the normal head and neck tissues[illegible]h and low Rin 1[illegible] compared with ages ≤40,>40 in the head and neck cancer of p- value 0.02[illegible]ere was a significant difference between the histological differentiation of the malignant tumor with p values of 0.001, when poor and well moderate was compared. [illegible]NCLSION Our data suggest that RIN1 plays an important role in head and neck tumor progression and that its expression will provide baseline data to facilitate identification of new molecular targeting therapeutics.
**Order of Authors:**	Roland Osei Saahene, PHD
	Precious Barnes, PhD
	F A Yeboah
	Elvis Agbo, PhD
	Du-Bois Asante, PhD
	Samuel Kofi Arhin, PhD
**Additional Information:**	
**Question**	**Response**
**Financial Disclosure**<br><br>Enter a financial disclosure statement that describes the sources of funding for the work included in this submission. Review the submission guidelines for detailed requirements. View published research articles from *PLOS ONE* for specific examples.<br><br>This statement is required for submission	No fundings

Powered by Editorial Manager® and ProduXion Manager® from Aries Systems Corporation

and **will appear in the published article** if the submission is accepted. Please make sure it is accurate.<br><br>**Unfunded studies**<br>Enter: *The author(s) received no specific funding for this work.*<br><br>**Funded studies**<br>Enter a statement with the following details:<br>• Initials of the authors who received each award<br>• Grant numbers awarded to each author<br>• The full name of each funder<br>• URL of each funder website<br>• Did the sponsors or funders play any role in the study design, data collection and analysis, decision to publish, or preparation of the manuscript?<br>• **NO** - Include this sentence at the end of your statement: *The funders had no role in study design, data collection and analysis, decision to publish, or preparation of the manuscript.*<br>• **YES** - Specify the role(s) played.<br><br>* typeset	
**Competing Interests**<br><br>Use the instructions below to enter a competing interest statement for this submission. On behalf of all authors, disclose any competing interests that could be perceived to bias this work—acknowledging all financial support and any other relevant financial or non-financial competing interests.<br><br>This statement is required for submission and **will appear in the published article** if the submission is accepted. Please make sure it is accurate and that any funding sources listed in your Funding Information later in the submission form are also declared in your Financial Disclosure statement.<br><br>View published research articles from *PLOS ONE* for specific examples.	The authors have declared that no competing interests exist.

<table>
<tr><td>

NO authors have competing interests

Enter: *The authors have declared that no competing interests exist.*

Authors with competing interests

Enter competing interest details beginning with this statement:

I have read the journal's policy and the authors of this manuscript have the following competing interests: [insert competing interests here]

* typeset
</td><td></td></tr>
<tr><td>

Ethics Statement

Enter an ethics statement for this submission. This statement is required if the study involved:

- Human participants
- Human specimens or tissue
- Vertebrate animals or cephalopods
- Vertebrate embryos or tissues
- Field research

Write "N/A" if the submission does not require an ethics statement.

General guidance is provided below. Consult the submission guidelines for detailed instructions. **Make sure that all information entered here is included in the Methods section of the manuscript.**
</td><td>The research protocol was approved by the Ethical Review Committee of the Cape Coast Teaching Hospital (REF: CCTHERC/RS/EC/2017/52) and the Committee on Human research, publications and Ethics of Kwame Nkrumah University of Science and Technology, School of Medical Sciences and Komfo Anokye Teaching Hospital (CHRPE/AP/609/18).</td></tr>
</table>

Powered by Editorial Manager® and ProduXion Manager® from Aries Systems Corporation

**Format for specific study types**<br><br>**Human Subject Research (involving human participants and/or tissue)**<br>• Give the name of the institutional review board or ethics committee that approved the study<br>• Include the approval number and/or a statement indicating approval of this research<br>• Indicate the form of consent obtained (written/oral) or the reason that consent was not obtained (e.g. the data were analyzed anonymously)<br><br>**Animal Research (involving vertebrate animals, embryos or tissues)**<br>• Provide the name of the Institutional Animal Care and Use Committee (IACUC) or other relevant ethics board that reviewed the study protocol, and indicate whether they approved this research or granted a formal waiver of ethical approval<br>• Include an approval number if one was obtained<br>• If the study involved *non-human primates*, add *additional details* about animal welfare and steps taken to ameliorate suffering<br>• If anesthesia, euthanasia, or any kind of animal sacrifice is part of the study, include briefly which substances and/or methods were applied<br><br>**Field Research**<br><br>Include the following details if this study involves the collection of plant, animal, or other materials from a natural setting:<br>• Field permit number<br>• Name of the institution or relevant body that granted permission	
**Data Availability**<br><br>Authors are required to make all data underlying the findings described fully available, without restriction, and from the time of publication. PLOS allows rare exceptions to address legal and ethical concerns. See the PLOS Data Policy and FAQ for detailed information.	Yes - all data are fully available without restriction

Powered by Editorial Manager® and ProduXion Manager® from Aries Systems Corporation

A Data Availability Statement describing where the data can be found is required at submission. Your answers to this question constitute the Data Availability Statement and **will be published in the article**, if accepted.<br><br>**Important:** Stating 'data available on request from the author' is not sufficient. If your data are only available upon request, select 'No' for the first question and explain your exceptional situation in the text box.<br><br>Do the authors confirm that all data underlying the findings described in their manuscript are fully available without restriction?	
**Describe where the data may be found in full sentences. If you are copying our sample text, replace any instances of XXX with the appropriate details.**<br><br>• If the data are **held or will be held in a public repository**, include URLs, accession numbers or DOIs. If this information will only be available after acceptance, indicate this by ticking the box below. For example: *All XXX files are available from the XXX database (accession number(s) XXX, XXX.).*<br>• If the data are all contained **within the manuscript and/or Supporting Information files**, enter the following: *All relevant data are within the manuscript and its Supporting Information files.*<br>• If neither of these applies but you are able to provide **details of access elsewhere**, with or without limitations, please do so. For example:<br><br>*Data cannot be shared publicly because of [XXX]. Data are available from the XXX Institutional Data Access / Ethics Committee (contact via XXX) for researchers who meet the criteria for access to confidential data.*<br><br>*The data underlying the results presented in the study are available from (include the name of the third party*	Available at University of Cape Coast Library

and contact information or URL).<br>• This text is appropriate if the data are owned by a third party and authors do not have permission to share the data.<br><br>* typeset	
Additional data availability information:	Tick here if the URLs/accession numbers/DOIs will be available only after acceptance of the manuscript for publication so that we can ensure their inclusion before publication.

Powered by Editorial Manager® and ProduXion Manager® from Aries Systems Corporation

Manuscript

Click here to access/download;Manuscript;Manuscript on expression of RIN1 in head and neck tumor.pdf

TITLE: EXPRESSION OF RAS AND RAB INTERACTOR 1 (RIN1) IN HEAD AND NECK TUMORS AT SELECTED HOSPITAL IN GHANA

Roland Osei Saahene[a], Precious Barnes[b], F. A. Yeboah[c], Elvis Agbo[d], Du-Bois Asante[e], Samuel Kofi Arhin[b]

[a]Department of Microbiology and Immunology, School of Medical Sciences, College of Health and Allied Sciences, University of Cape Coast, Ghana

[b]Department of Physician Assistant Studies, College of Health and Allied Sciences School of Allied Health Sciences, University of Cape Coast, Ghana

[c]Department of Molecular Medicine, School of Medical Sciences[illegible]llege of Health Sciences, Kwame Nkrumah University of Science and Technology, Ghana

[d]Elvis Agbo, Department of Human Anatomy, Histology and Embryology, College of Medicine, Jinggangshan University, Ji'an City, China

[e]Department of Forensic Sciences, School of Biological Sciences, College of Agricultural and Natural Sciences, University of Cape Coast, Ghana

Dr. Precious Barnes: Corresponding author
Email address: precious.barnes@ucc.edu.gh

ABSTRACT:

BACKGROUND: Head and neck tumor (HNT) are tumor of the paranasal sinuses, the salivary glands and the upper aerodigestive tract. RIN1 is a Ras effector protein regulating epithelial cell properties and has been implicated in a number of cancers.

METHOD: The aim of this study was to investigate the expression of RIN1 in head and neck tumor. RIN1 expression was analyzed using quantitative real-time PCR (qRT-PCR) and immunohistochemical staining on tissue samples from a consecutive series of 150 head and neck tumor patients who underwent tumor resections between 2014 and 2020.

RESULTS: The relationship between RIN1 expressions, clinicopathological factors, was investigated. qRT-PCR results showed that the RIN1 mRNA expression was low in tumor tissue samples than in t[illegible] RIN1expression were low as compared with the normal head and neck tissues. [illegible]gh and low Rin 1 was compared with ages ≤40,>40 in the head and neck cancer of p- value 0.02. [illegible]re was a significant difference between the histological differentiation of the malignant tumor with p values of 0.001, when poor and well moderate was compared.

CONCLSION: Our data suggest that RIN1 plays an important role in head and neck tumor progression and that its expression will provide baseline data to facilitate identification of new molecular targeting therapeutics.

INTRODUCTION:

Head and neck cancers are cancers of the salivary glands, the paranasal sinuses (spaces around the nose lined with cells that make mucus), the nasal cavity, and the upper aerodigestive tract which includes the oral cavity, pharynx (throat), and larynx (voice box) with about 90% being squamous cell carcinomas (Kumar, et al, 2010; Pfeiffer et al., 2011). The anatomical sites affected result in the loss of important functions such as speech, taste, smell and swallowing.

World Health Organization (WHO) in 2002 estimated an average of 600 000 new cases of HNC and 350 000 deaths each year worldwide. Oral cancer was identified as the commonest HNC with about 389 000 new cases annually followed by laryngeal and pharyngeal cancers with 160 000 and 65 000 new cases respectively. According to the WHO summary report update on human papillomavirus and related cancers in Ghana (September 15 2010), about three hundred and thirty (330) new cases of oral cancer are reported annually with approximately one hundred and fifty (150) deaths occurring out of this worldwide incident.

The development of HNC is a multistep process requiring the accumulation of genetic

alterations, influenced by factors such as tobacco and alcohol intake, viral infections, poor nutrition and others against a background of heritable susceptibility to mutagens.

The RIN1 gene is located on chromosome 11q13.2 (Shuster et al., 2000) and consists of a coding region of 2352bp constituting 783 amino acids. Its expression in cells is restricted, being predominantly expressed in forebrain neurons most notably in the hippocampus, cortex, striatum and amygdala (Dhaka et al., 2003). Moderate levels are expressed in mouse testes (Dhaka *et al*., 2003), hematopoietic cells, mammary epithelial cells and perhaps other epithelial cells of other tissues (Hu et al., 2005, Milstein et al., 2007). The expression level is however undetected in mouse embryonic fibroblast (Dzudzor et al., 2010), the midbrain and forebrain (Dhaka *et al*., 2003). The restricted expression is regulated by cis-acting elements located at the 5'region of the gene (Dzudzor et al., 2010).

RIN1 functions through two downstream pathways involved in maintenance of epithelial properties namely the activation of Rab5 (Tall et al., 2001) and Abl family tyrosine kinases (Hu et al., 2005). By activating ABL tyrosine kinases, RIN1 blocks the cytoskeletal rearrangements associated with cell dissociation and migration. The Ras and Rab interactor 1 (RIN1) is a multidomain Rab5 guanine nucleotide exchange factor that plays an important role in Ras-activated endocytosis and growth factor receptor trafficking (Tall et al., 2001). RIN1 is thus a Ras effector protein with its Ras binding domain (RBD) localized in its carboxyl terminal region. This region also binds 14-3-3 proteins which act as a negative regulator of membrane localization of RIN1 proteins (Wang et al., 2002). RIN1 interacts with the effector domain of activated Ras through competitive inhibition of Raf which is the best characterized of the Ras effector proteins. Raf is a mitogen activated protein kinase kinase kinase (MAP KKK) and its interaction with activated Ras gets it activated to set of the MAP kinase cascade which transduces signals resulting in changes in protein activity and gene expression.

RIN1 signaling through Rab5 proteins promotes endocytosis of EGFR and several other growth factor receptors. After activation, the receptor is degraded within multivesicular bodies (MVB) and lysosomes, an important cellular process known as receptor down-regulation for signal attenuation. Alterations in receptor trafficking and signal attenuation have been associated with carcinogenesis (Katzmann, et al 2002, Dikic and Giordano, 2003, Husnjak, and Dikic, 2006). Of the many cellular systems that are impacted by EGFR signaling, the activation of the Rasdependent extracellular-regulated kinase (ERK) cascade appears to have the most pronounced effect on the proliferative response to epidermal growth factors (EGF). While some of the genomic alterations correlate with genes known to be important in head and neck squamous cell carcinomas such as p16, p53, and Cyclin D1, many of the specific genes such as RIN1 are still unknown. This presents the need for research into possible genes such as RIN1 which encodes a protein regulating epithelial cell properties, and whose expression is found altered in cancerous breast and colorectal epithelial cells (Milstein et al., 2007, Senda et al, 2006). The purpose of this study is thus to provide useful information about the possible role of RIN1 in head and neck tumors and which may provide baseline data to facilitate the identification of new molecular targeting therapeutics.

MATERIALS AND METHODS

Study Design

The study protocol was a retrospective in design, which consisted of laboratory analysis of archived head and neck tumor tissue samples from 2014 to 2020. The data were accessed for the research purposes on 20th March, 2021 from Cape Coast Teaching Hospital and Komfo Anokye Teaching Hospital.

Patient Tissue Samples

Paraffin-embedded blocks of samples (archival tissue specimens) from patients with histologically HNT specimens were collected from the pathology laboratory at Cape Coast Teaching Hospital and komfo Anokye teaching Hospital in Ghana. Histologic diagnosis and differentiation were evaluated by a pathologist according to the World Health Organization classification system. The clinicopathologic features are shown in Table 1and 2. Prior to the initiation of this study, the research was approved by the Institutional Ethics Committee of the Cape Coast Teaching Hospital and the Committee of Human Research, Publications and Ethics at kwame Nkrumah University of Science and Technology, School of Medical Sciences & Komfo Anokye Teaching Hospital, the patients informed consent was waived by the ethics committees. The research protocol was approved by the Ethical Review Committee of the Cape Coast Teaching Hospital (REF: CCTHERC/RS/EC/2017/52) and the Committee on Human research, publications and Ethics of Kwame Nkrumah University of Science and Technology, School of Medical Sciences and Komfo Anokye Teaching Hospital (CHRPE/AP/609/18).

IMMUNOHISTOCHEMISTRY

The expression of RIN1 was analyzed by immunohistochemistry (IHC). The goat anti-RIN1 (Biosynthesis Biotechnology Co LTD, Beijing China) antibody was used. 4um formalin fixed paraffin embedded (FFPE) mounted sections were deparaffinized, rehydrated and rinsed three times in distilled water. After heat antigen retrieval and cooling in citrate buffer solution, they were incubated with 5% bovine serum albumin (BSA) at 37ºC for 30 minutes following three times phosphate-buffered saline (PBS) rinses. The slides were incubated at 4ºC with primary antibody RIN 1(1:500 dilution) overnight followed by incubation with Biotinylated anti-rabbit

immunoglobulin G (IgG) and SABC at 37°C for 30 minutes each. The slides were thoroughly rinsed in between incubations with PBS three times. Next, they were developed for 5 minutes in 3,3'-diaminobenzene (DAB) and counter-stained with Mayer's hematoxylin at room temperature. Subsequently, they were washed with distilled water, HCL in ethanol and PBS. They were then incubated in PBS at 37°C for 40 minutes to 1 hour. Finally, the slides were dehydrated in graded series of ethanol and xylene, cover slipped and mounted under the microscope. The brown and blue stained cells viewed under the microscope represent positive and negative cells respectively. The immunoreaction was subjectively assessed by experienced pathologists. RIN1-expressing cells were scored semiquantitatively according to the number of positive-staining cells and the staining intensity. cytoplasmic immunostaining in the tumor cells was considered positive staining. The immunohistochemical results of RIN1 were grouped into 2 categories: low expression (0 to 1+) and high expression (2+ to 3+).

TOTAL RNA EXTRACTION, REAL-TIME PCR

The total RNA was isolated from the tumor specimens with a TRIzol reagent (Takara, Otsu, Japan) according to the manufacturer's instructions. Reverse transcription was performed using 0.5 mg total RNA from each sample. Real time quantitative polymerase chain reaction (RT-qPCR) was performed using the SYBR Green PCR Master Mix (Takara). The sequences of the primer pairs were as follows: RIN1 (forward), 5'-GGCAGCAGAGGAGTAGCTTGA-3'; RIN1 (reverse), 5'-GCTTGCTGGCGCTAAAAGG-3'; GAPDH (forward), 5'-ATAGCACAGCCTGGATAGCAACGTAC-3'; and GAPDH (reverse), 5'-CACCTTCTACAATGAGCTGCGTGTG-3'. The experiments were repeated in triplicate. The relative levels of gene expression were represented as ΔCt = Ct of RIN1 – Ct of GAPDH, and the fold change of gene expression was computed using the 2–ΔΔCt method.

STATISTICAL ANALYSIS

All statistical analyses were performed using SPSS 17.0 for Windows (SPSS, Chicago, IL). Significant level was set at 0.05. For qualitative variables, proportions and percentages was use to summarize the data. The association between RIN1 expression and HNT ~~patient~~ clinicopathologic features was evaluated using the $\chi 2$ test.

Table 1.1 Association between RIN 1 and Clinico-pathologic Characteristics in Patients with Head and Neck Malignant Tumor.

Variables	Total (n=98)	Low RIN1 (n=71)	High RIN1 (n=27)	cOR (95%CI)	p-value
**Age (years)**					
≤40	33(33.7)	19(26.8)	14(51.9)	1	
>40	65(66.3)	52(73.2)	13(48.1)	0.34(0.13-0.85)	**0.021**
**Sex**					
Male	62(63.2)	45(63.4)	17(63.0)	1	
Female	36(36.7)	24(36.6)	12(37.0)	1.32(0.54-3.22)	0.537
**Grade**					
Well	36(36.7)	24(33.8)	12 (44.4)	1	
Moderate	46(46.9)	30(42.3)	16(59.3)	1.07(0.42-2.68)	0.891
Poor	16(16.3)	15(21.1)	1(3.7)	0.13(0.02-1.13)	0.045
**Tumor site**					
Eye	1(1.0)	1(1.4)	0(0.0)	1	
Oral cavity	36(36.7)	4(33.8)	12(44.4)	8.33(0.28-244.07)	0.219
Nasal Cavity	28(28.6)	22(31.0)	6(22.2)	0.87(0.03-23.91)	0.933
Larynx	7(7.1)	5(7.0)	2(7.4)	1.36(0.04-46.65)	0.863
Mandible	14(14.3)	10(14.1)	4(14.8)	1.29(0.04-37.98)	0.884
Nasopharynx	11(11.2)	9(12.7)	2(7.4)	0.79(0.02-25.90)	0.894
Salivary gland	1(1.0)	0(0.0)	1(3.7)	9.00(0.10-831.85)	0.341
Tumor stage					
I/II	51(42.4)	17(63.0)	34(47.9)	1	
III/IV	47(48.0)	11(37.0)	36(52.1)	1.64(0.67-3.99)	0.279

Table 1.2 Association between RIN 1 and Clinico-pathologic Characteristics in Patients with Head and Neck Benign Tumor.

Variables	Total (n=52)	Low RIN1 (n=24)	High RIN1 (n=28)	cOR (95%CI)	p -value
**Age**					
≤40	34(65.4)	15(62.5)	19(67.9)	1	
>40	18(34.6)	9(37.5)	9(32.1)	0.79(0.25-2.48)	0.686
**Sex**					
Male	28(53.8)	15(62.5)	13 (46.4)	1	
Female	24(46.2)	9(37.5)	15(53.6)	1.92(0.63-5.84)	0.249
**Tumor site**					
Oral cavity	18(34.6)	11(45.8)	7(25.0)	1	
Nasal Cavity	11(21.1)	4(16.7)	7(25.0)	2.75(0.58-12.97)	0.201
Larynx	2(3.8)	0(0.0)	2(7.1)	7.67(0.32-183.02)	0.208
Mandible	18 (34.6)	8(33.3)	10(35.7)	1.96(0.52-7.41)	0.319
Nasopharynx	3(5.7)	1(4.2)	2(7.1)	3.14(0.24-41.51)	0.385

RESULT

THE LEVEL OF EXPRESSION OF RAS AND RAB INTERACTOR 1 (RIN1) IN HEAD AND NECK TUMOURS

Figure 1.1 shows the RIN1 expression levels in the normal head and neck tissues compared with the paired head and neck cancerous tissues and figure 1.1 shows the immunohistochemistry expressions of RIN1 in tumor tissue RIN1 is silenced in Head and Neck tumor. The expression of RIN1 in 150 head and neck tumors were compared with commercial positive controls by immunohistochemistry. The relative expression of RIN1 protein was significantly lower in the tumor compared to control samples [control: 5.45 (3.83-9.30) vs case: 1.80 (0.88-2.70); p-value = 8.802×10^{-5}]. In both the control and tumors, RIN1 protein was localized in the cytoplasm. The immunostaining results recorded for the tumors ranged from negative, weak staining, moderate staining and strong staining (Figure 1.1).

Using logistic regression analysis, we determined the clinico-pathologic characteristics associated with high RIN1 expression in Head and Neck Malignant Tumor. Of the factors include in the analysis, only age was significantly associated with RIN1 expression. Patients with advanced age (>40 years old) presented with significantly lower odds ratio for expressing high RIN1 levels [OR = 0.34, 95% CI (0.13-0.85), p-value = 0.021] (Table 1.1). A similar risk stratification was performed for patients with benign Head and Neck Tumor. However, no statistically significant association between clinico-pathologic characteristics and relative expression of RIN1 was found (Table 1.2).

DISCUSSION

The Site of RIN1 in the Head and Neck Tumor

Understanding the expression and the localization of RIN1 in head and neck tumor is of great value in developing a novel therapeutic strategy. RIN1 initiates Rab5 GTPases, which control endocytosis of cell-surface receptors, and also it initiates ABL non-receptor tyrosine kinases activities to participates in actin cytoskeleton remodeling.

RIN1 is involve in tumor metastasis and development but to our knowledge, there is no study on the level of RIN1 in head and neck tumors especially in Ghana. In this research, we investigated the level of expression of RIN1 in head and neck tumors and the normal tissues. We also explored the correlation between the levels of RIN1 expression and clinical features such as gender, age and the anatomical site in head and neck tumor.

The RIN1 gene exhibits variable localization in different tumors. From this present study, RIN1 was localized at the cytoplasm in the head and neck tumor tissues (Micrograph 1.1 and 1.2), this is in accordance to a study by Senda et al (2007). From their study, RIN1 was found in the cytoplasm of colorectal cancer. The presence of RIN1 in the cytoplasm indicates that it is involved in the cell membrane signaling pathway to maintain the epithelial integrity (Milstein et al., 2007). RIN1 has the ability to inactivate uncontrolled cell growth which is initiated by Rab5 but this mechanism is inhibited when RIN1 is present at the cytoplasm (Pendergast, 2002).

The Level of Expression of RIN1 in Head and Neck Tumors and Normal Tissues

The level of expression of RIN 1 varies in tumors. From this present study, the level of expression of RIN1 is reduced or silenced in head and neck tumor tissues but was highly expressed in the normal head and neck tissues (Micrograph 1.1 and 1.2 and fig 1.1). This is similar to a study by Milstein et al (2007), they observed that the level of RIN1 was silence in breast tumor tissues

but the level of expression of RIN1 increased in tumors such as gastric adenocarcinoma (Yu et al., 2012), colorectal cancer (Senda et al., 2007), non–small cell lung cancer (Wang et al., 2012), and bladder urothelial carcinoma (Shan et al., 2012). RIN1 transcript levels from head and neck tumors and normal head and neck tissues were also determined with real time polymerase chain reaction (RT-PCR) in Fig 1.1. Subsequently, we examined low levels of RIN1 protein in head and neck tumor tissues. This was similar to a study by Marc et al; (2007) who found low expression of mRNA RIN 1 in breast tumor tissues when they were compared with normal breast tissues.

RIN1 is very crucial in the proliferative response to epidermal growth factors (EGF) and therefore low levels of RIN1 affects cell proliferation which is associated with the response to epidermal growth factor. The activation of Rab5 by RIN1 is therefore important in the control of the signaling pathway of EGFR. Therefore, when RIN1 is silenced, EGFR signaling will not be controlled by the Rab5 and this will result in a high abnormal proliferation which will favour tumorgenesis.

Patients with advanced age (>40 years old) presented with significantly lower odds ratio for expressing high RIN1 levels [OR = 0.34, 95% CI (0.13-0.85), p-value = 0.021] (Table 1.1). 15 of the patients had poor differentiated tumor with low expression of rin1 out of 16 poor differentiated tumors (Table 1.1). There are two potential mechanisms that cause reduction in RIN1 expression. These mechanisms are DNA methylation of the RIN1 promoter and also transcriptional repression which result in high levels of SNAI1 (Barrallo et al., 2005).

The Effect of SNAI1 on RIN1 Expression

SNAI1 over expression contribute to RIN1 silencing in tumors. SNAI1 is a transcriptional repressor of genes that ensures the integrity of epithelial cell properties such as differentiation, cell division and cell to cell adhesion (Cano et al., 2000). Tumors with low RIN1 levels has a

higher level of SNAI1 than their normal tissues and therefore elevated levels of SNAI1 contribute to the silencing of RIN1 in tumors (Marc et al., 2007).

The Effect of DNA Methylation on RIN1 Expression

The level of RIN1 expression is reduced as a result of DNA methylation. DNA methylation takes place at the CpG dinucleotides in the promoter region of RIN1 (Baylin et al., 2006). It therefore results in the mobilization of chromatin remodeling complexes in cells which causes a reduction in RIN1 expression (Baylin et al., 2006).

CONCLUSION

In summary, our results indicate that RIN1 is a tumor suppressor gene in HNT, making RIN1 an attractive target for future cancer therapeutics.

Ethical approval and consent to participate

The Institutional Review Board of the Kwame Nkrumah University of Science and Technology gave their approval for the study (Ethical Clearance ID: CHRPE/AP/609/18) and also the Ethical Review Board of Cape Coast Teaching Hospital gave approval for the commencement of the research (Ethical Clearance ID: CCTHERC/RS/EC/2017/52).

Funding

The study was funded by University of Cape Coast

Availability of data and materials

The datasets used will be deposited in the archives of Kwame Nkrumah University of Science and Technology Library and is available from the corresponding author on reasonable request.

Authors' contributions

PB, FAY, ROS-conceived the study, contributed to the study design, data collection and analysis, manuscript preparation. FAY, BA, MA, JW, YAA- contributed to the preparation and finalization of the manuscript and data analysis and also contributed in the laboratory analysis.

Competing interests

The authors confirmed that there is no competing interest.

ACKNOWLEDGMENTS

This work was supported by grants from University of Cape Coast. Our profound gratitude to the staff of Immunology laboratory especially the head of the Immunology Department

REFERENCES

Barrallo-Gimeno A, Nieto MA. (2005) The Snail genes as inducers of cell movement and survival: implications in development and cancer. Development.61,132:3151.

Baylin SB, Ohm JE. (2006) Epigenetic gene silencing in cancer - a mechanism for early oncogenic pathway addiction? Nat Rev Cancer.20, 6:107–16..

Cano A, Perez-Moreno MA, Rodrigo I, et al. (2000) The transcription factor snail controls epithelial-mesenchymal transitions by repressing E-cadherin expression. Nature cell biology. 2, 76–83.

Dhaka, A, Costa, R.M, Hu, H, et al. (2003). The RAS effector RIN1 modulates the formation of aversive memories. J.Neurosci. 23, 748-757.

Dikic I, Giordano S (2003) Negative receptor signalling**.** Curr Opin Cell Biol. 15(2):128-35.

Dzudzor, B, Huynh, L, Thai, M, et al. (2009). Regulated expression of the Ras effector Rin1 in forebrain neurons, Mol. Cell. Neurosci. 22, 1328–1343.

Hu H, Bliss JM, Wang Y, Colicelli J. (2003) RIN1 is an ABL tyrosine kinase activator and a regulator of epithelial cell adhesion and migration. Curr Biol 15:815–23.

Husnjak K, Dikic I (2006) EGFR trafficking: parkin' in a jam. Nat Cell Biol. 8(8):787-8.

Katzmann DJ, Odorizzi G, Emr SD (2002): Receptor downregulation and multivesicular-body sorting. [Nature reviews Molecular cell biology]. Vols. 3(12):893-910.

Kumar A, Petri ET, Halmos B, Boggon TJ (2010): Structure and clinical relevance of the epidermal growth factor receptor in human cancer. [J. Clin. Oncol]. Vols. 26:1742–1751.

Marc Milstein, Chelsea K. Mooser, Hailiang Hu, et al. (2007) RIN1 is a Breast Tumor Suppressor Gene Cancer Res. 67, 11510–11516.

Milstein, M., Mooser,C.K., Hu,H., et al. (2007). RIN1 is a breast tumor suppressor gene. Cancer Res 67, 11510-11516.

Pendergast AM. (2002) The Abl family kinases: mechanisms of regulation and signaling. Adv Cancer Res.85, 51-100.

Pfeiffer, Brenda Y. Hernandez, et al (2011) J Clin Oncol. 10; 29(32): 4294–4301.

Senda K, Goi T, Hirono Y, et al. (2007) Analysis of RIN1 gene expression in colorectal cancer. Oncol Rep.17, 1171-1175.

Stetak, A, Hoier, EF, Croce A, et al (2006) Cell fate-specific regulation of EGF receptor trafficking during Caenorhabditis elegans vulval development EMBO J. 25, 2347–2357

Shan GY, Zhang Z, Chen QG, et al. (2012) Overexpression of RIN1 associates with tumor grade and progression in patients of bladder urothelial carcinoma. Tumour Biol. 33, 847-855.

Shuster MI, Han L, Le Beau MM, et al (2000): A consistent pattern of RIN1 rearrangements in oral squamous cell carcinoma cell lines supports a breakage-fusion-bridge cycle model for 11q13 amplification. [Genes Chromosomes Cancer]. Vols. 28(2):153-163.

Tall GG, Barbieri MA, Stahl PD, Horazdovsky B. F., (2001). Ras activated endocytosis is mediated by the Rab5guanine nucleotide exchange activity of RIN1. Dev Cell; 1: 73–82.

Thai M, Ting PY, McLaughlin J, et al. (2011) ABL fusion oncogene transformation and inhibitor sensitivity are mediated by the cellular regulator RIN1. Leukemia. 25, 290-300.

Wang Q, Gao Y, Tang Y, et al. (2012) Prognostic significance of RIN1 gene expression in human non-small cell lung cancer. Acta Histochem.114, 463-468.

Wang,Y, Waldron,R.T, Dhaka,A, et al (2002). The RAS effector RIN1 directly competes with RAF and is regulated by 14-3-3 proteins. Mol. Cell Biol. 22, 916-926.

W3enig BM, Cohen J-M. (2009) General principles of management of head and neck cancer: general principles of head and neck pathology. In: Harrison LB, Sessions RB, Hong WK, editors. Head and neck cancer: a multidisciplinary approach, 3rd ed. Philadelphia: Lippincott, William & Wilkins. 3–50.

Yu HF, Zhao G, Ge ZJ, et al. (2012) High RIN1 expression is associated with poor prognosis in patients with gastric adenocarcinoma. Tumour Biol.33, 1557-1563

 Click here to access/download;Figure;Fiqures for RIN1.docx

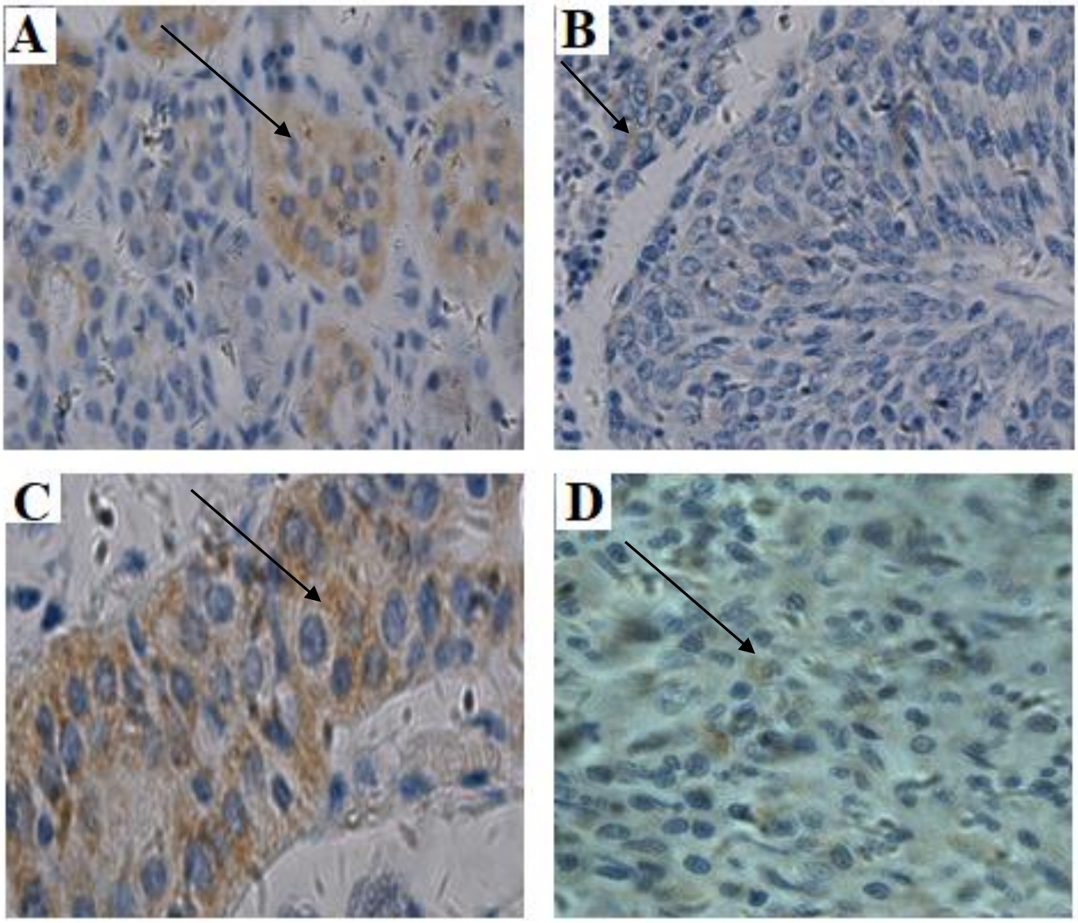

Micrograph 1.1: RIN1 expression levels were upregulated in the control head and neck tissues (A and C) compared with the paired head and neck cancerous tissues (B and D) X400.

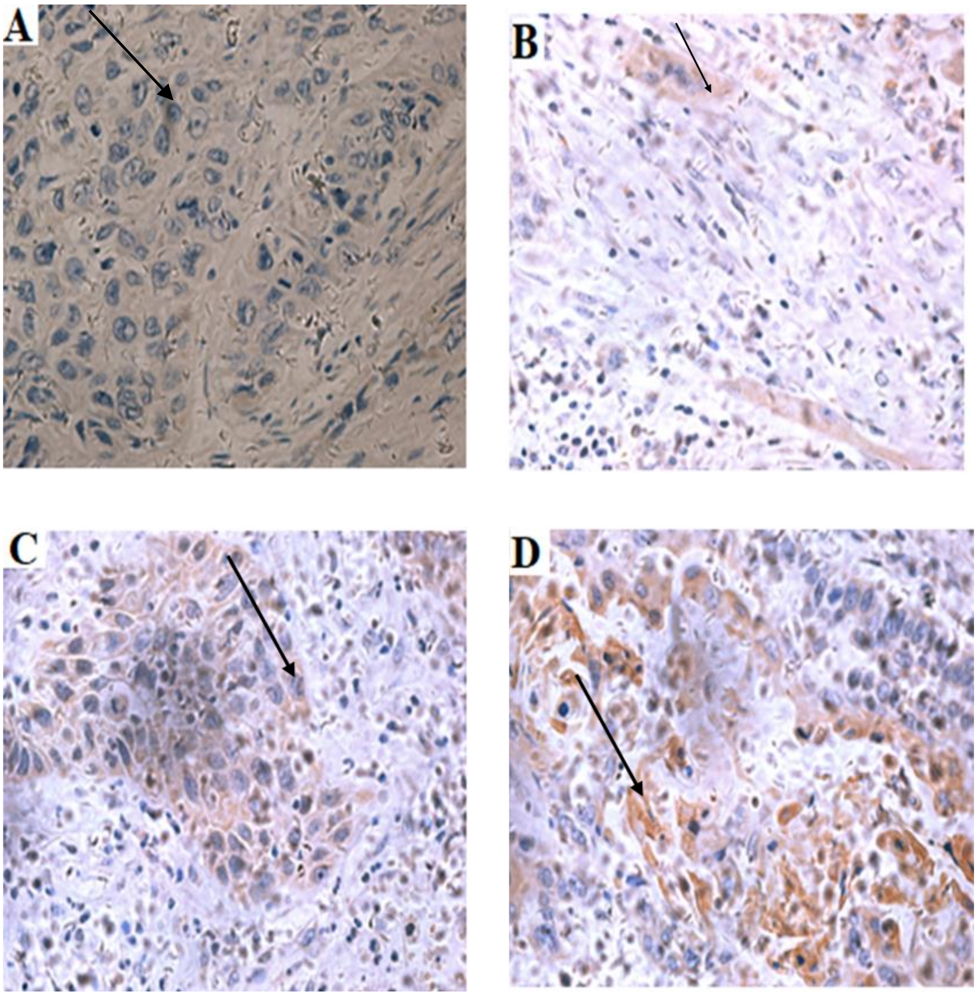

Micrograph 1.2 Immunohistochemistry expressions of RIN1 in tumour tissue. A is at tumour tissue with negative RIN1 expression (0); B is a tumour tissue with a weak expression (1+); C is a tumour tissue with moderate expression of RIN1 (2+); D is a tumour tissue with highly expression of RIN1 (3+). The data was produced from 3-5 fields per slide. Magnification ×400.Used arrows to point out the histological differences.

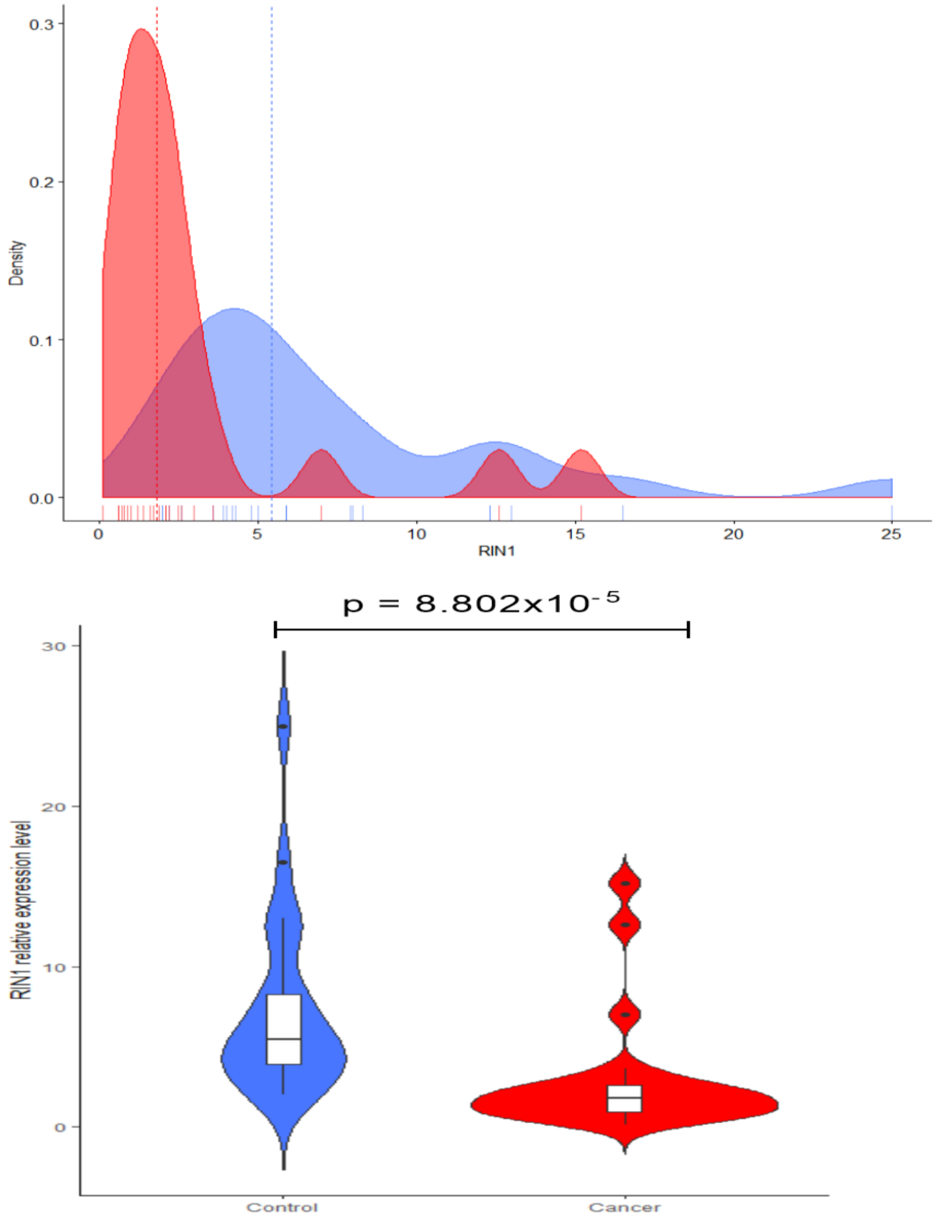

Figure 1.1 A graph showing the level of expression of RIN1 in the control and the tumour tissues by real-time polymerase chain reaction.
