# Supplementary material for: Expression of RAS and RAB interactor 1 (RIN1) in head and neck tumors at selected hospital in Ghana
Source: PLoS One. 2024 Apr 18;19(4):e0301295. doi: 10.1371/journal.pone.0301295 (PMC11025731; doi:10.1371/journal.pone.0301295)
Supplement: Attachment — Submitted filename: EXPRESSION OF RAS AND RAB INTERACTOR I.docx [file pone.0301295.s001.docx]

EXPRESSION OF RAS AND RAB INTERACTOR I (RIN1) IN HEAD AND NECK TUMORS AT SELECTED HOSPITAL IN GHANA.

| Section | Comments |
| --- | --- |
| Title Page | Item C   - The Department of Molecular Medicine is situated in the School of Medicine and Dentistry. The name, School of Medical Sciences has long been changed. |
| Abstract |  |
| Background | Line 1   - Subject verb agreement. Head and neck was presented in the singular as ‘tumor’ and the verb used was ‘are’. - This must be revised as the definition covers a number of sites which exceeds one unit.   Line 3   - The sentence ‘… has been implicated in a number of cancers’ is vague. - For a manuscript of this value, the suggested or intended implication, whether positive or negative should be stated to set the foundation for the research. |
| Methods | Line 5   - The sentence ‘…RIN1 expression was analyzed using quantitative real-time PCR…’ needs to be revised as PCR is not a tool for analyzing in itself.   Line 6   - The samples were not selected from consecutive series but rather a retrospective study which relied on tissue blocks. This must be corrected and revised. - The 150 represents tissue blocks prepared from samples obtained from patients rather than the presentation that it was 150 neck and tumour patients. - The 150 tissue blocks were selected from different facilities.   Line 8   - Restatement of the aim/objective. Rephrase and concentrate on the standard format for presentation of results.   Line 9   - Grammatical errors and incoherence in sentence construction. Example, ‘… low in tumour tissue samples than in t RIN1….’   Line 10   - Aside control slides used to guide immune-staining, there were no samples labelled as normal which were used in comparative analysis with the case samples. - ‘high and low Rin 1…’ check the sequence and flow.   Line 11   - The sentence is in disarray and does not communicate relevant information to be consumed as results.   Lines 12-13   - Sentence disarray. Revise. |
| Introduction |  |
|  | Line 1   - In the abstract section, authors used the tag ‘head and neck tumour’ but in the first line of the introduction, this was presented as ‘head and neck cancers’. Authors must be more specific with terminology.   Line 7   - Estimates of HNC cases were quoted from a 2002 WHO reference document. Between the years 2002 to date, data on HNC cases will have sharp variations from that quoted from 20 years ago.   Line 11   - WHO summary report as mentioned was not referenced. - There is no linkage with the introduction of papillomavirus in the line of argument with RIN1.   Line 14-16   - No reference for the kind of information stated.   Line 17   - Wrong positioning of the reference   Line 27   - States ‘Ab1 family tyrosine kinases’ which was re-stated as ‘ABL tyrosine kinases’ in Line 28. - Authors must be consistent with the presentation of technical terms.   Line 35   - The sentence flow is incoherent. Revise.   Line 37   - Sentence requires correction.   Line 39   - EGFR was introduced and never defined on first use.   Line 45   - Check the sentence.   Line 53   - Check the sentence.   Overall summary   - The introduction presents hurriedly put together information from literature with general lack of organization, structure and mechanical accuracy. The sentence constructions are ladened with grammatical and subject verb agreement errors which impacts negatively on the general expression. - The introduction must be reorganized and the information presented in a coherent manner to provide a strong foundation for the research. |
| Materials & Methods | Line 57   - Sentence correction.   Lines 59-61   - Authors indicate samples were collected from the Cape Coast and Komfo Anokye Teaching Hospitals respectively but the numbers accessed for evaluation and the final numbers selected for analysis were not presented. - An algorithm which provides a framework on the numbers selected right to the final numbers must be clearly presented.   Line 61   - Komfo Anokye Teaching Hospital was spelt with lower case letters.   Line 62   - Makes reference to the WHO classification system with no reference.   Line 64   - Reference to Tables 1 and 2 without detailed presentation of what the clinicopathologic details entail under methods is inappropriate.   Lines 64-68   - Presents information about ethical clearance   Lines 68-72   - Presents same information on ethical clearance   Line 74   - The micro symbol (µ) has been presented with the letter ‘u’   Line 79   - RIN1 which has been presented in this format in varied sections of the document is presented as RIN 1. - ‘biotinylated’ was spelt with a capital ‘B’.   Line 80   - SABC has been presented without any definition.   Line 81   - PBS has been presented without any definition.   Line 83   - HCL has been presented without any definition.   Lines 87-91   - The same set of information presented in repetitive manner. - Emphasis should be made of the point that semiquantitative estimates cannot be used in place of quantitative estimates.   Line 100   - Statements were made about delta Ct with no details on the threshold values.   Line 101   - What does 2-ΔΔCt method mean?   Line 104   - States significant levels was set at 0.05 which is an error in the statement of significance levels as this is supposed to be <0.05. - None of the parameters presented are quantitative so it begs the question how authors are setting p-values.   Line 105 |
| Tables 1 and 2 | The tables are presented under the methods section and not results as the case should be.   - Why are the tables labelled as 1.1 and 1.2 respectively? - Without any indication from the methods section, the samples have been categorized as head and neck malignant tumour (Table 1.1) and head and neck benign tumour (Table 1.2). - How many of the malignant tumours were selected from the study sites? - How many of the benign samples were collected from the study sites? |
| Table 1.1 | - What categorization was used to classify the malignant tumours as Low RIN1 and High RNI1? - If the dependent variables is categorized on ‘low’ and ‘high’, how will the logistic regression analysis be interpreted? - No footnotes have been presented for the table to explain the type of analysis done and the considerations taken for such analysis. - Under the variable sections, ‘Grade’ which has not been defined was analyzed against the dependent variable which is also categorized on the bases of low and high. This must be explained with clarity else presents a possible error of double estimation. |
| Table 1.2 | - The benign tumours were categorized as low RIN1 and high RIN1 with no indication of what defines the categorization. - For a dependent variable being classified as low and high, how will authors interpret the logistic regression analysis? |
| Results | Lines 107-110   - It is very difficult to make meaning of the sentence based on the structure and grammatical errors.   Line 110   - States that expression of RIN1 in 150 head and neck tumours were compared with commercial positive controls by immunohistochemistry. - Authors must appreciate the fact that the inclusion of a control slide in a staining process is not to infer comparison for all samples evaluated but rather to authenticate the validity of the staining method and process. - Therefore, beyond staining of the malignant and benign tumours, no comparison with controls can be inferred. This must be corrected within the context of the sentences and statements made.   Lines 1112-113   - Authors quote quantitative values for the first time without any indication on how these values were arrived at. - A p-value of 8.802 x 10^-5^ should give authors a cue that there is an inherent analytical error in whatever analysis they intended to conduct.   Line 118   - Authors state that logistic regression was used to analyze clinicopathological characteristics associated with RIN1 expression in malignant and benign tumours: - Authors should have noted from the statement that the expression of RIN1 should have been evaluated as present or absent irrespective of the strength categorizations in the positive cases. - Categorization of the dependent variable as low and high may not support the interpretations made.   Overall:   - The results should be carefully evaluated and separated into clear categories which will support the statistical analysis which will be adopted. |
| Discussion | Line 131   - The sentence ‘RIN1 is involve in tumour…’ should be corrected.   Line 134   - States that correlation was explored in the study. As a matter of fact, no results summary on correlation was carried out in the study.   Line 138   - Referencing is wrong   Line 144   - Check how RIN 1 is written in comparison to its presentation in the write-up.   Line 146   - The micrographs are labelled ‘micrograph 1.1 and 1.2’ respectively which is in error according to the labelling of figures.   Line 161-163   - There is no foundational basis within the scope of work done to assert to such a fact.   Line 166   - RIN1 is presented as ‘rin1’   Line 168   - No foundational basis for such assertions.   Line 177   - No basis for the assertion.   The discussion has been given paragraph headings which is not the prescribed format for publication presentation. This needs revision and organization to present a coherent justification to support the aims and assertions of the study. |
| Conclusion | The conclusion as presented cannot be supported by any analysis presented in the study. |
| References | - The referencing style is not uniform. - The references are in disarray, example, two author publications are referenced with et al., (Barallo et al., 2005) - The reference ‘W3enig’ appears nowhere in-text. - Most of the reference list have been presented with et al instead of a full presentation of the author list. |
