# Supplementary material for: Expression of RAS and RAB interactor 1 (RIN1) in head and neck tumors at selected hospital in Ghana
Source: PLoS One. 2024 Apr 18;19(4):e0301295. doi: 10.1371/journal.pone.0301295 (PMC11025731; doi:10.1371/journal.pone.0301295)
Supplement: Attachment — Submitted filename: RESPONSE TO EDITOR AND REVIEWERS.docx [file pone.0301295.s003.docx]

**RESPONSE TO EDITOR AND REVIEWERS’ COMMENTS**

Thank you for your extensive review and insightful comments and suggestions on our manuscript. We appreciate the attention to detail and your dedication to enhancing the quality of our work.

We have carefully considered your comments and corrected all relevant grammatical, citation and referencing errors. Also all table and figure labelling errors have been rectified.

**Comment**: Why categorization into benign and malignant?

Response: Benign is not cancerous and therefore can not be graded or staged. Hence, their characteristics can not be compared malignant tumours (that are staged and graded), but the expression levels (either upregulated or down regulated) of biomarkers can be compared with each other.

**Comment**: The categorization does not support a logistic regression analysis.

**Response**: We acknowledge that logistic regression is typically applied to continuous predictors, and in our study, we opted for categorical representations of Age, Sex, Grade, and Tumor site based on the clinical relevance of these categories. Our research objectives were centered around exploring the associations between these categorical variables and RIN1 expression levels. The categorical representation allowed us to explore the relationships between these factors and RIN1 expression in a straightforward and clinically relevant manner.

**Comment**: Under the variable sections, ‘Grade’ which has not been defined was analyzed against the dependent variable which is also categorized on the bases of low and high. This must be explained with clarity else presents a possible error of double estimation. (Table 1)

**Response**:

**Comment**: For a dependent variable being classified as low and high, how will authors interpret the logistic regression analysis? (Table 2)

**Response**: In the logistic regression analysis, our goal was to assess the probability of the binary outcome variable (RIN1 Expression; as low or high). Specifically, we examined how the independent variables (Age, Sex, Grade, Tumor site and Tumor stage) relate to the likelihood of observing the outcome in the 'high' category compared to the 'low' category.

Furthermore, the odds ratio associated with each independent variable provided a quantifiable measure of the impact of that variable on the likelihood of the either of the binary outcome (high or low RIN1 expression). For example, an odds ratio greater than 1 suggests an increased likelihood of the 'high' outcome, while an odds ratio less than 1 suggests a decreased likelihood.
